# Non-TZF Transcriptional Activator AtC3H12 Negatively Affects Seed Germination and Seedling Development in Arabidopsis

**DOI:** 10.3390/ijms23031572

**Published:** 2022-01-29

**Authors:** Hye-Yeon Seok, Taehyoung Kim, Sun-Young Lee, Yong-Hwan Moon

**Affiliations:** 1 Institute of Systems Biology, Pusan National University, Busan 46241, Korea; seokhyeon@pusan.ac.kr (H.-Y.S.); symoonlee@pusan.ac.kr (S.-Y.L.); 2 Department of Integrated Biological Science, Pusan National University, Busan 46241, Korea; kth3245@naver.com; 3 Department of Molecular Biology, Pusan National University, Busan 46241, Korea

**Keywords:** Arabidopsis, AtC3H12, CCCH zinc finger, non-TZF, seed germination, seedling development, transcriptional activator

## Abstract

CCCH zinc finger proteins are a large protein family and are classified as either tandem CCCH zinc finger (TZF) or non-TZF proteins. The roles of TZF genes in several plants have been well determined, whereas the functions of many non-TZF genes in plants remain uncharacterized. Herein, we describe biological and molecular functions of AtC3H12, an Arabidopsis non-TZF protein containing three CCCH zinc finger motifs. AtC3H12 has orthologs in several plant species but has no paralog in Arabidopsis. *AtC3H12*-overexpressing transgenic plants (OXs) germinated slower than wild-type (WT) plants, whereas *atc3h12* mutants germinated faster than WT plants. The fresh weight (FW) and primary root lengths of *AtC3H12* OX seedlings were lighter and shorter than those of WT seedlings, respectively. In contrast, FW and primary root lengths of *atc3h12* seedlings were heavier and longer than those of WT seedlings, respectively. AtC3H12 was localized in the nucleus and displayed transactivation activity in both yeast and Arabidopsis. We found that the 97–197 aa region of AtC3H12 is an important part for its transactivation activity. Detection of expression levels and analysis of Arabidopsis transgenic plants harboring a *P*_AtC3H12_::*GUS* construct showed that *AtC3H12* expression increases as the Arabidopsis seedlings develop. Taken together, our results demonstrate that AtC3H12 negatively affects seed germination and seedling development as a nuclear transcriptional activator in Arabidopsis. To our knowledge, this is the first report to show that non-TZF proteins negatively affect plant development as nuclear transcriptional activators.

## 1. Introduction

Zinc finger proteins constitute a large group of protein families categorized to different types, such as C2H2, C2C2, C2HC, C2C2C2C2, C2HCC2C2, and CCCH, on the basis of the number and order of cysteine (Cys) and histidine (His) residues that bind to a zinc ion [1,2]. They participate in various biological processes, including transcription, apoptosis, and protein assembly [1,3,4].

CCCH zinc finger proteins, which are broadly found in yeast and higher eukaryotes, are determined based on the CCCH zinc finger motif, which consists of three Cys residues and one subsequent His residue [5]. Sixty-eight CCCH zinc finger protein genes have been recognized in the Arabidopsis (*Arabidopsis thaliana*) genome, whereas 67 genes have been recognized in rice (*Oryza sativa*) [5]. Of the 68 CCCH zinc finger proteins in Arabidopsis, 26 are tandem CCCH zinc finger (TZF) proteins with two tandem CCCH zinc finger motifs, and 42 are non-TZF proteins with one or more than two CCCH zinc finger motifs [6].

To date, the functions of TZF genes have been well studied in several plants. In Arabidopsis, *AtTZF3*/*Oxidation-related Zinc Finger 2* (*AtOZF2*) is engaged in abscisic acid (ABA) response and the salt stress response [7]. *AtTZF4*/*SOMNUS* (*SOM*) negatively regulates seed germination by regulating ABA and gibberellic acid (GA) metabolic genes [8]. *AtTZF6*/*PEI1* plays an important role during embryogenesis [9]. *AtTZF9* mediates immune response triggered by pathogen-associated molecular patterns [10]. *AtTZF10*/*Salt-inducible Zinc Finger 2* (*AtSZF2*) and *AtTZF11*/*AtSZF1* function in the salt stress response [11]. In rice, *OsTZF1* confers abiotic stress tolerance and negatively modulates leaf senescence under stress conditions [12]. *OsC3H10* modulates the expression of stress-responsive genes and improves drought tolerance [13]. *PvC3H72*, a TZF gene in switchgrass (*Panicum vigatum*), is involved in cold tolerance by controlling the ICE1-CBF-COR regulon and ABA-responsive genes [14]. Overexpression of *PeC3H74*, a TZF gene in moso bamboo (*Phyllostachys edulis*), in Arabidopsis renders the transgenic plants drought tolerant [15]. Similarly, overexpression of *PdC3H17* in poplar (*Populus deltoides* × *P. euramericana*) enhances drought tolerance [16].

In contrast, the functional roles of non-TZF genes have not been well studied compared to TZF genes. In Arabidopsis, *KHZ1*/*AtC3H36* and *KHZ2*/*AtC3H52* act not only in stress responses and aging but also in flowering [17]. *AtC3H17* pleiotropically modulates development and the salt stress response [6,18]. In rice, the *leaf and tiller angle increased controller* (*OsLIC*) is known to participate in architecture regulation mediated by brassinosteroid signaling [19]. *IbC3H18*, a non-TZF gene in sweet potato (*Ipomoea batatas*), is associated with abiotic stress tolerance [20]. *Male Fertility 30a* (*BcMF30a*) and *BcMF30c* in Chinese cabbage (*Brassica campestris*) are involved in pollen development [21,22]. AtC3H59/ZFWD3 interacting with deSUMOylating isopeptidase1 (Desi1) participates in seed germination, seedling development, and seed development [23]. Despite an increasing number of studies on non-TZF genes, the roles of many non-TZF genes in plants still remain unclear.

CCCH zinc finger proteins are known as RNA-binding proteins and associated with post-transcriptional regulation of mRNA. In Arabidopsis, AtTZF1, AtC3H14, AtC3H15/AtCDM1, cleavage and polyadenylation specificity factor 30 (AtCPSF30), and HUA1 have been revealed to bind to RNA [4,24,25,26]. The TZF motif of AtTZF1 plays a key role in binding to RNA [25]. AtC3H3 possesses ribonuclease function, binding to RNA [27].

In addition to its role in RNA metabolism, most recent studies demonstrated that CCCH zinc finger proteins also modulate transcription. Two Arabidopsis TZF proteins, AtC3H14 and AtC3H15/AtCDM1, display transactivation activity and bind to both DNA and RNA in vitro [4]. OsLIC activates transcription via its EELR domain [19]. Recently, AtC3H17, PdC3H17, IbC3H18, and PvC3H72 have also been revealed to be transcriptional activators [6,14,16,20].

Well-conserved transactivation motifs have been recognized on transcriptional activators in plants. In CCCH zinc finger proteins, the EELR motif in OsLIC has been identified as a transactivation motif [19]. Similarly, the EELR-like motif of AtC3H17 is responsible for transactivation activity [6]. The AHA motif in the homeodomain–leucine zipper (HD-Zip) I family proteins, including Homeobox 1 (AtHB1), AtHB7, AtBH12, and AtHB13, also functions in transcriptional activation [28]. Additionally, the LWSY and EDLL motifs serve as transactivation motifs in Apetala 2 (AP2)/ ethylene-responsive factor (ERF) transcription factors, such as related to AP2.12 (RAP2.12), RAP2.2, and AtERF98 [29,30]. Recently, EELL-, VDDG-, and LWSY-like motifs in AtERF73/hypoxia-responsive ERF 1 (HRE1), an Arabidopsis AP2/ERF transcription factor, have been reported as transactivation motifs [31].

In this study, we selected a previously uncharacterized Arabidopsis non-TZF protein, AtC3H12 showing strong transactivation activity and characterized its biological and molecular functions. We demonstrated that AtC3H12 negatively affects seed germination and seedling development as a nuclear transcriptional activator. Our findings will enable us to expand our knowledge of the functions of non-TZF proteins as transcriptional regulators.

## 2. Results

### 2.1. AtC3H12 Has Three CCCH Zinc Finger Motifs

**Figure 1:**
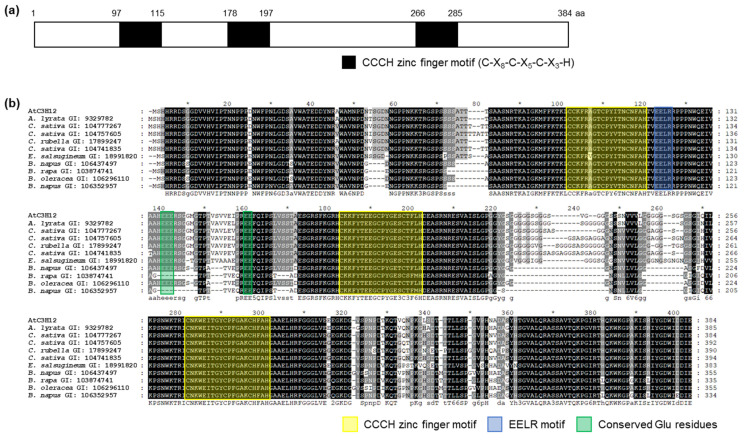
Protein domain of AtC3H12 and multiple alignment among AtC3H12 and its orthologs. (**a**) CCCH zinc finger motifs of AtC3H12. CCCH zinc finger motifs are represented as black boxes. (**b**) Multiple-sequence alignment was performed using the ClustalW2 program with amino acid sequences of AtC3H12 and its orthologs. Three conserved CCCH zinc finger motifs are annotated as yellow boxes. Black-, dark-gray-, and light-gray-shaded amino acids represent 100%, 80%, and 60% conservation rate, respectively. Blue and green boxes represent the EELR motif and conserved Glu residues, respectively. * display positions which have a single, fully conserved residue. The GI number of each protein sequence is as follows: AtC3H12, 840128; *A*. *lyrata*, 9329782; *C*. *sativa*, 104777267; *C*. *sativa*, 104757605; *C*. *rubella*, 17899247; *C*. *sativa*, 104741835; *E*. *salsugineum*, 18991820; *B*. *napus*, 106437497; *B*. *rapa*, 103874741; *B*. *oleracea*, 106296110; *B*. *napus*, 106352957.

To isolate non-TZF protein(s) that act as transcription factors, we screened non-TZF gene(s) that show high transactivation activity in yeast and selected AtC3H12 (At1g32360) for further study (data not shown). AtC3H12, a non-TZF protein, has three CCCH zinc finger motifs that are designated as C-X_8_-C-X_5_-C-X_3_-H (Figure 1a). In order to identify homologous genes of *AtC3H12*, BLASTP analysis was performed. AtC3H12 has no paralog in Arabidopsis but has several orthologs in other plant species, such as *Arabidopsis lyrata*, *Camelina sativa*, *Capsella rubella*, *Eutrema salsugineum*, *Brassica napus*, *Brassica rapa*, and *Brassica oleracea* (data not shown). Multiple-sequence alignment showed that amino acid sequences were highly conserved among AtC3H12 and its orthologs, especially in the N-terminus, CCCH zinc finger motifs, and C-terminus (Figure 1b).

### 2.2. AtC3H12 Is Negatively Associated with Seed Germination and Seedling Development

**Figure 2:**
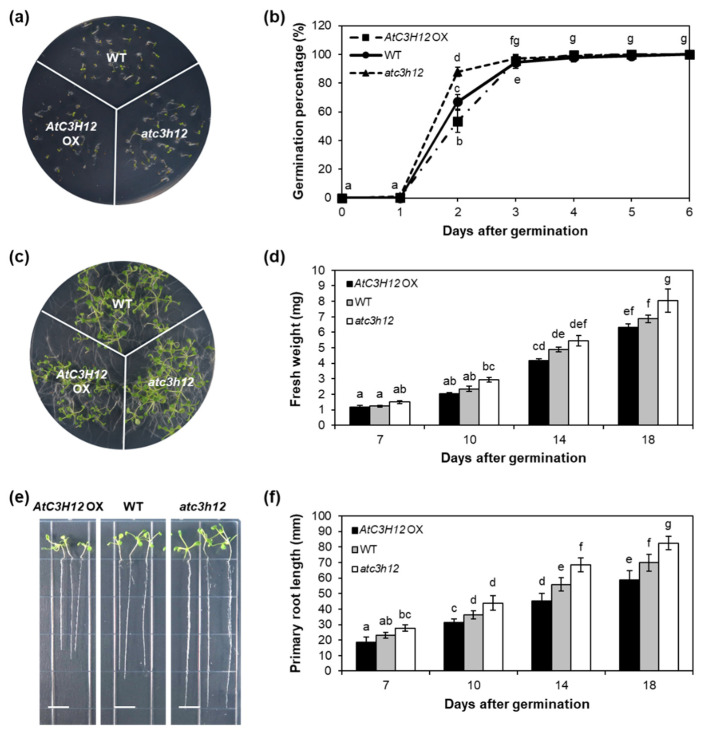
Seed germination and seedling development of *AtC3H12* OXs and *atc3h12* mutants. (**a**) Four-day-old WT, *AtC3H12* OX-7, and *atc3h12* seedlings grown on Murashige and Skoog (MS) agar plates under short-day (SD) conditions. (**b**) Germination percentage of WT, *AtC3H12* OX-7, and *atc3h12* mutants measured at specified times after sowing on MS agar plates. Germination was verified by radicle protrusion. Error bars display standard deviation (*n* = 20). (**c**) Fourteen-day-old WT, *AtC3H12* OX-7, and *atc3h12* seedlings grown on MS agar plates under SD conditions. (**d**) Fresh weight of WT, *AtC3H12* OX-7, and *atc3h12* seedlings grown on MS agar plates at 7, 10, 14, and 18 DAG. Error bars display standard deviation (*n* = 5). (**e**) Elongation of primary roots of WT, *AtC3H12* OX-7, and *atc3h12* seedlings at 14 DAG. The white lines indicate scale bar = 1 cm. (**f**) Primary root lengths of WT, *AtC3H12* OX-7, and *atc3h12* seedlings grown on MS agar plates under SD conditions were measured at 7, 10, 14, and 18 DAG. Error bars display standard deviation (*n* = 10). In (**b**,**d**,**f**), different letters display significant differences (*p* < 0.05).

To study the biological functions of *AtC3H12*, we generated *AtC3H12*-overexpressing transgenic plants (OXs) and selected homozygous *atc3h12* mutants (Figures S1 and S2). First, seed germination was analyzed. Seeds of *AtC3H12* OX plants germinated significantly slower than those of WT plants, whereas *atc3h12* mutants germinated faster than WT (Figure 2a,b and Figure S3a,b). In particular, the germination percentage of *AtC3H12* OXs and *atc3h12* mutants was significantly lower and higher, respectively, than that of the WT 2 days after germination (DAG) (Figure 2b and Figure S3b). However, the final germination percentage did not differ among *AtC3H12* OXs, *atc3h12* mutants, and WT (Figure 2b and Figure S3b). During seedling development from 7 to 18 DAG, *AtC3H12* OX seedlings were smaller and lighter than WT seedlings (Figure 2c,d and Figure S3c,d). In contrast, *atc3h12* seedlings were heavier and larger than WT seedlings (Figure 2c,d). In particular, the increase in fresh weight (FW) from 10 to 14 DAG in *atc3h12* mutants was higher than that of WT (Figure 2d). In addition, we found that the primary root length of *AtC3H12* OXs was shorter than that of WT, whereas the primary root length of *atc3h12* mutants was longer than that of WT from 7 to 18 DAG (Figure 2e,f and Figure S3e,f). These results indicate that *AtC3H12* has a negative role in seed germination and seedling development.

We investigated the flowering time of *AtC3H12* OXs and *atc3h12* mutants to determine whether *AtC3H12* functions at the reproductive developmental stage as well as at the vegetative developmental stage. To determine flowering time, the number of rosette leaves was counted at bolting under long-day conditions. There was no considerable difference in flowering time among WT, *AtC3H12* OXs, and *atc3h12* mutants (Figure S4), indicating that *AtC3H12* may not be engaged in the regulation of flowering time.

### 2.3. AtC3H12 Protein Is Localized in the Nucleus

**Figure 3:**
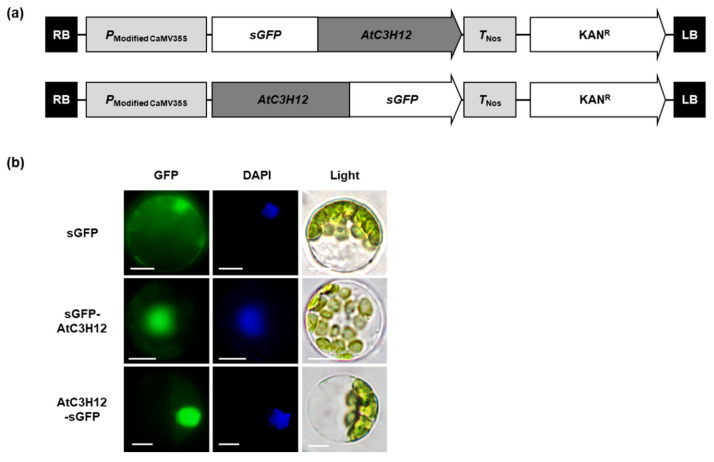
Subcellular localization of AtC3H12. (**a**) Schematic maps of the sGFP-fused, full-length ORF of AtC3H12 constructs. (**b**) Subcellular localization of AtC3H12 protein investigated by transient expression of sGFP-AtC3H12 and AtC3H12-sGFP constructs in Arabidopsis protoplasts. Left, GFP signal; middle, 4′,6-diamidino-2-phenylindole (DAPI) staining; right, light microscopic picture. The white lines indicate scale bar = 10 μm.

We studied the subcellular localization of the AtC3H12 protein in Arabidopsis protoplasts using N-terminal (sGFP-AtC3H12) and C-terminal (AtC3H12-sGFP) synthetic green fluorescent protein (sGFP)-fused AtC3H12 constructs to determine the potential molecular function of the protein (Figure 3a). As a result, GFP signals of sGFP-AtC3H12 and AtC3H12-sGFP constructs were exclusively observed in the nucleus (Figure 3b), indicating that AtC3H12 may exert its functions in the nucleus.

### 2.4. The 97–197 aa Region of AtC3H12 Is Responsible for Its Transactivation Activity

**Figure 4:**
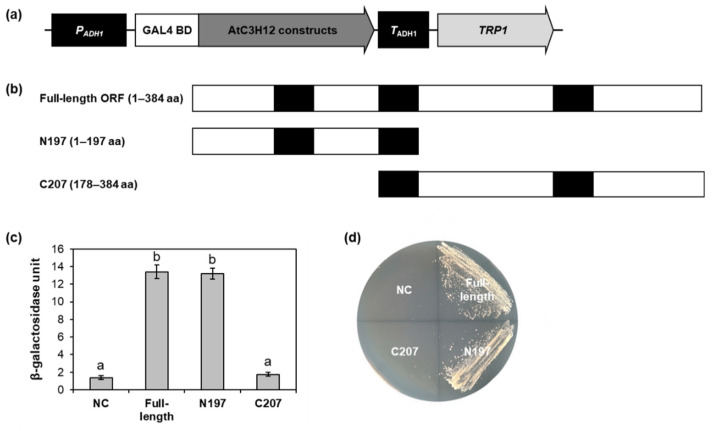
Transactivation activity assay of AtC3H12 in yeast. (**a**) Schematic map of the GAL4 BD-fusion vector for transactivation activity assay in yeast. (**b**) Schematic maps of full-length ORF, N197, and C207 of AtC3H12 for transactivation activity assay. (**c**) Quantitative β-galactosidase ONPG assay. β-Galactosidase activities were measured to quantify the transactivation activities. Error bars display standard deviation (*n* = 3). Different letters display significant differences (*p* < 0.05). (**d**) Yeast growth assay. Yeast transformants were grown on SD media lacking Trp and Ura (SD-Trp/-Ura). In (**c**,**d**), empty pBD-GAL4 vector was used for a negative control. NC, negative control.

To determine the transactivation domain of AtC3H12, AtC3H12 was divided into two regions: the N-terminal 1–197 aa region (N197), in which the first and the second CCCH zinc finger motifs were contained, and the C-terminal 178–384 aa region (C207), in which the second and third CCCH zinc finger motifs were present (Figure 4b). The full-length open reading frame (ORF), N197, and C207 were separately cloned into pBD-GAL4 to generate GAL4 DNA-binding domain (BD)-AtC3H12 fusion constructs (Figure 4a) and transformed into yeast. In a quantitative β-galactosidase orthonitrophenyl-β-D-galactopyranoside (ONPG) assay and yeast growth assay, N197 showed transactivation activity in yeast as well as full-length ORF (Figure 4c,d and Figure S5a).

**Figure 5:**
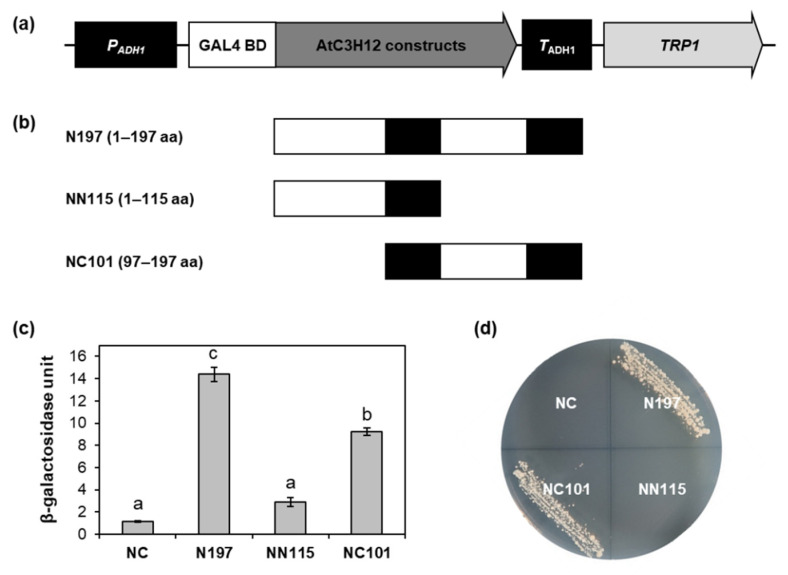
Transactivation activity assay of N197 of AtC3H12 in yeast. (**a**) Schematic map of the GAL4 BD-fusion vector for transactivation activity assay in yeast. (**b**) Schematic maps of N197, NN115, and NC101 of AtC3H12 for transactivation activity assay. (**c**) Quantitative β-galactosidase ONPG assay. β-Galactosidase activities were measured to quantify the transactivation activities. Error bars display standard deviation (*n* = 3). Different letters display significant differences (*p* < 0.05). (**d**) Yeast growth assay. Yeast transformants were grown on SD-Trp/-Ura. In (**c**,**d**), empty pBD-GAL4 vector was used for a negative control. NC, negative control.

To narrow down the transactivation domain, N197 of AtC3H12 was divided into two regions, the 1–115 aa region (NN115) and 97–197 aa region (NC101), and cloned into pBD-GAL4 (Figure 5a,b). In the ONPG assay and yeast growth assay using the yeast transformants containing GAL4 BD-AtC3H12 fusion constructs, NC101 showed transactivation activity, whereas NN115 displayed no activity. (Figure 5c,d and Figure S5b).

**Figure 6:**
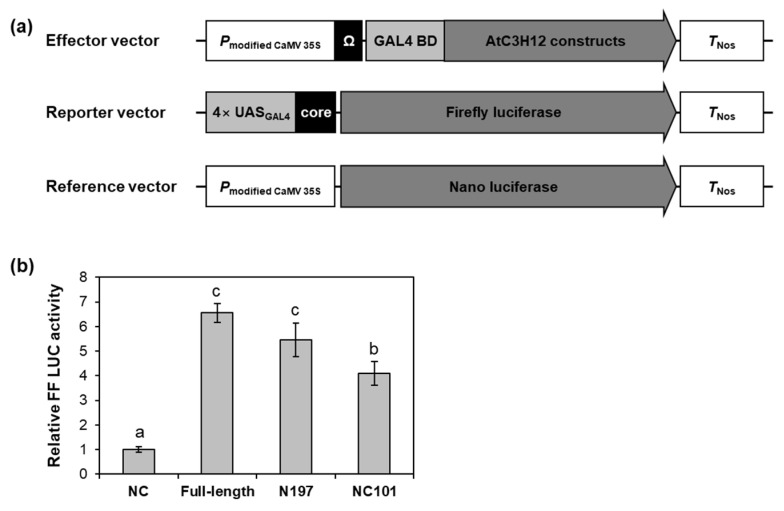
Transactivation activity assay of AtC3H12 in Arabidopsis protoplasts. (**a**) Schematic maps of the effector vector, reporter vector, and reference vector for transactivation activity assay. (**b**) Relative firefly luciferase (FF LUC) activities of full-length ORF, N197, and NC101 of AtC3H12 in Arabidopsis protoplasts. The reference vector was used for the normalization of transformation efficiency. The empty effector vector was used for a negative control. Normalized FF LUC activity of negative control was set to 1. Error bars display standard deviation (*n* = 3). Different letters display significant differences (*p* < 0.05).

To verify the transactivation activity of AtC3H12 in Arabidopsis, effector vectors, in which the full-length ORF, N197, or NC101 of AtC3H12 were linked to GAL4 BD, were generated and introduced into Arabidopsis protoplasts (Figure 6a). Transient expression of each effector vector along with reporter vector demonstrated that all of the full-length ORF, N197, and NC101 showed transactivation activity (Figure 6b). These results are compatible with the data acquired from yeast (Figure 4c and Figure 5c) and suggest that NC101 is responsible for the transactivation activity of AtC3H12.

Next, we compared the amino acid sequences of NC101 of AtC3H12 and the corresponding regions of AtC3H12 orthologs. Amino acid sequences of the regions were highly conserved among the NC101 and orthologs, especially in the EELR motif and Glu residues (Figure 1b). It has previously been reported that the EELR motif is important for transactivation activity [19]. In addition, acidic amino acid residues, such as Glu and Asp, are also involved in transactivation activity [32]. These results suggest that the EELR motif and conserved Glu residues in NC101 of AtC3H12 might play a key role in its transactivation activity.

### 2.5. Expression Levels of AtC3H12 during Development and in the Organs of Arabidopsis

**Figure 7:**
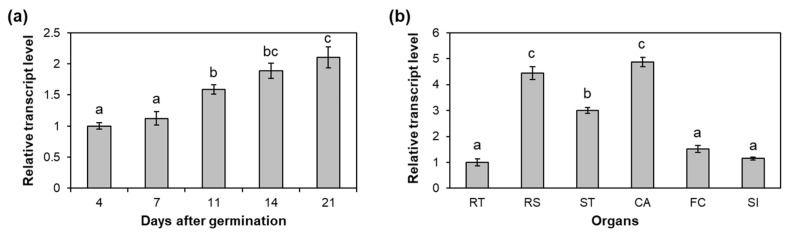
Temporal and spatial expression patterns of *AtC3H12*. (**a**) Quantitative RT-PCR (RT-qPCR) analysis of *AtC3H12* in 4-, 7-, 11-, 14-, and 21-day-old WT seedlings grown under SD conditions. Transcript level in 4-day-old seedlings was set as 1. (**b**) RT-qPCR analysis of *AtC3H12* in organs of 50-day-old WT plants grown under long-day conditions. Transcript level in RT was set as 1. RT, roots; RS, rosette leaves; ST, stems; CA, cauline leaves; FC, floral clusters; SI, siliques. *GAPc* was used for an endogenous control gene. At least two biological replicates showed similar results. Error bars display standard deviation (*n* = 3). Different letters display significant differences (*p* < 0.05).

We examined the expression patterns of *AtC3H12* in different developmental stages and organs by quantitative RT-PCR (RT-qPCR). The *AtC3H12* transcript level was slightly elevated as the seedlings developed from 4 to 21 days after germination (DAG) (Figure 7a). In mature plants, cauline leaves and rosette leaves showed a higher level of *AtC3H12* transcripts than other organs, such as roots, stems, floral clusters, and siliques (Figure 7b).

**Figure 8:**
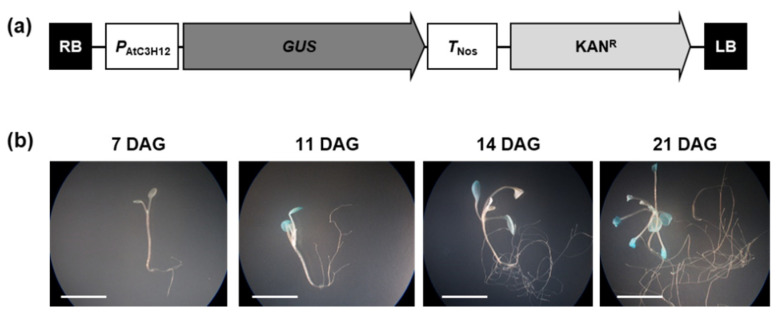
Analysis of the promoter activity of *AtC3H12*. (**a**) Schematic map of *P*_AtC3H12_::*GUS* for GUS assay. (**b**) Histochemical assay of GUS activities in transgenic plants harboring *P*_AtC3H12_::*GUS* at different developmental stages grown under SD conditions. Three independent T_1_ lines showed similar results, with one shown here. The white lines indicate scale bar = 1 cm.

To visualize the expression patterns of *AtC3H12*, transgenic plants harboring the *P*_AtC3H12_::*GUS* construct were generated and analyzed by a histochemical β-glucuronidase (GUS) assay (Figure 8a). First, we compared the promoter activities of *AtC3H12* with and without the 559-bp 5′ UTR (Figure S6a). As a result, the *AtC3H12* promoter with 5′ UTR showed insignificant GUS activity (Figure S6b). Thus, we used *P*_AtC3H12_::*GUS* transgenic plants without the 5′ UTR for further experiments. The histochemical GUS assay revealed that GUS activity was detected mainly in the cotyledons and the leaves of 7, 11, 14, and 21 DAG seedlings, and the activity increased as the seedlings grew (Figure 8b), supporting the result that *AtC3H12* expression increases as plants develop (Figure 7a).

## 3. Discussion

CCCH zinc finger proteins are classified into two groups, TZF and non-TZF proteins [6]. Although non-TZF genes have been recently studied in several plant species, many still remain uncharacterized. Herein, we studied the functions of the Arabidopsis non-TZF gene, *AtC3H12*.

AtC3H12 has three CCCH zinc finger motifs (Figure 1a). Our BLASTP analysis showed that *AtC3H12* has orthologs in several plant species, but no paralog in Arabidopsis (Figure 1b), indicating that it is a unique gene in Arabidopsis. Our phenotype analysis using *AtC3H12* OXs and *atc3h12* mutants showed that *AtC3H12* plays important roles in seed germination and seedling development (Figure 2 and Figure S3). AtC3H12 was localized in the nucleus and showed transactivation activity via its 97–197 aa region (Figure 3, Figure 4, Figure 5 and Figure 6), demonstrating that AtC3H12 has pleiotropic effects during Arabidopsis vegetative development by transactivating downstream genes.

At the beginning of the study of the CCCH zinc finger proteins, the proteins were recognized as RNA-binding proteins participating in post-transcriptional regulation, including AtTZF1, AtC3H14, AtC3H15/AtCDM1, AtCPSF30, and HUA1 in Arabidopsis [4,24,25,26]. CCCH zinc finger proteins, such as AtC3H17, OsLIC, PdC3H17, IbC3H18, and PvC3H72, have also been characterized as transcriptional regulators [6,14,16,19,20]. However, in spite of functional characterization studies of CCCH zinc finger proteins as transcriptional regulators, activation or repression motifs/domains have been identified in only limited CCCH zinc finger proteins. OsLIC has an EELR domain as a transactivation domain, and the EELR motif in the EELR domain has been well conserved among orthologs of OsLIC [19]. AtC3H17 has an EELR-like motif, which consists of EE(D/E)AL(K/R) [6]. Our study identified the 97–197 aa region of AtC3H12 as a transactivation domain (Figure 5 and Figure 6). The EELR motif and Glu residues in the region are well conserved among AtC3H12 and its orthologs (Figure 1), showing that the EELR motif and Glu residues may play important roles in the transactivation activity of AtC3H12.

To reveal the biological function of *AtC3H12* in Arabidopsis development, we generated *AtC3H12* OX transgenic plants and obtained *atc3h12* T-DNA-inserted mutants (Figures S1 and S2). Phenotypic analysis showed that *AtC3H12* OXs germinated slower than WT, while *atc3h12* mutants germinated faster than WT (Figure 2a,b and Figure S3a,b). Moreover, *AtC3H12* OX seedlings were smaller, and primary root length was shorter than WT seedlings, whereas *atc3h12* seedlings were larger and primary root length was longer than WT seedlings (Figure 2c–f and Figure S3c–f). These results suggest that *AtC3H12* negatively influences seed germination and seedling development in Arabidopsis. However, *AtC3H12* OXs and *atc3h12* mutants showed no significant differences in flowering time (Figure S4). This is the first report to show that a non-TZF protein negatively affects plant development as a nuclear transcriptional activator.

Several CCCH zinc finger genes participate in plant development in different ways. In Arabidopsis, overexpression of *AtC3H17* enhances seed germination, seedling development, and seed development [6], and *AtC3H59*/*ZFWD3* also positively affects those processes, interacting with the PPPDE family protein Desi1 [23]. Similar to *AtC3H12*, overexpression of *AtC3H14*, an Arabidopsis TZF gene, resulted in defective cell elongation and dwarfism [33]. In rice, *OsLIC* is known to be involved in architecture regulation by the antagonistic function of *Brassinazole-Resistant 1* (*BZR1*) [34]. These reports demonstrate that appropriate plant development is orchestrated by positive and negative developmental regulations. It is suggested that *AtC3H12* might participate in the fine-tuning of development by negative regulation, together with other positive regulators in Arabidopsis. Recently, it has been reported that *AtC3H12* has a repressing effect on root hair density and root hair length depending on phosphorus availability [35]. It can be a clue to explain the function of *AtC3H12*. To explain how *AtC3H12* negatively regulates seed germination and seedling development, further studies are required for identification of the downstream target genes of *AtC3H12*.

**Figure 9:**
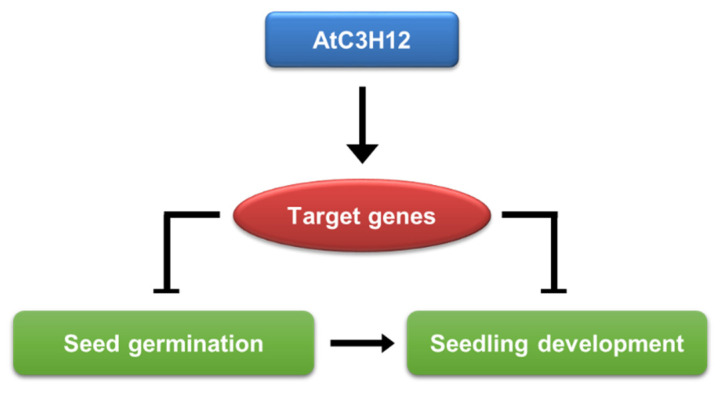
A functional model of the role of AtC3H12 in Arabidopsis development.

Collectively, our data propose that AtC3H12 containing three CCCH zinc finger motifs acts as a nuclear transcriptional activator to regulate the transcription of genes that negatively modulate seed germination and seedling development in Arabidopsis (Figure 9).

## 4. Materials and Methods

### 4.1. Arabidopsis Growth

Arabidopsis plants used in this research were of the Columbia (Col-0) ecotype. Arabidopsis seeds were prepared, germinated, and grown as previously described [23].

### 4.2. Multiple-Sequence Alignment

BLASTP analysis was conducted using NCBI BLAST (https://blast.ncbi.nlm.nih.gov/Blast.cgi, accessed on 12 August 2020). ClustalW2 (https://ebi.ac.uk/Tools/msa/clustalo, accessed on 12 August 2020) was used for multiple-sequence alignment.

### 4.3. Plasmid Construction

To generate constructs for subcellular localization, the full-length ORF of *AtC3H12* was cloned into pFGL1283 and pFGL1292 in frame with N-terminal and C-terminal sGFP, respectively [36]. To clone constructs for GUS assay, a 282-bp upstream region from the transcriptional start site of *AtC3H12*, with and without 559-bp 5′ UTR, was fused to the *GUS* gene [6]. To generate vectors for *AtC3H12* overexpression, the full-length ORF of *AtC3H12* was inserted into pFGL1434, including the modified CaMV *35S* promoter and N-terminal-fused HA tag [36].

To clone constructs for transactivation activity analysis in yeast, full-length ORF and partial fragments of AtC3H12 were cloned into the pBD-GAL4 in frame with GAL4 BD. To generate vectors for the transactivation assay in Arabidopsis protoplasts, full-length ORF and partial fragments of AtC3H12 were fused to GAL4 BD under the control of the modified CaMV *35S* promoter [31].

Primers for cloning are shown in Table S1.

### 4.4. Transgenic Plants and T-DNA-Inserted Mutants

The constructs were transformed into *Agrobacterium tumefaciens* strain GV3101 (pMP90) using the freeze–thaw method [37] and then introduced into WT Arabidopsis using the floral-dipping method [38]. Transgenic plants were selected on MS agar plates containing kanamycin (50 μg/mL). The T_3_ homozygous lines were used for further experiments.

T-DNA-inserted *atc3h12* mutant, SALK_011253 (*atc3h12*) was provided by the Salk Institute Genomic Analysis Laboratory.

### 4.5. Protoplast Transformation

The isolation and polyethylene glycol-mediated transformations of Arabidopsis protoplasts were conducted in accordance with Yoo et al. [39].

### 4.6. Analysis of the Transactivation Activity in Yeast

Yeast strain YD116 [40] was transformed using the Frozen-EZ Yeast Transformation II^TM^ Kit (Zymo Research Corp., Irvine, CA, USA), in accordance with the manufacturer’s instructions.

Quantitative β-galactosidase assay, β-galactosidase filter assay, and yeast growth assay were conducted as previously described [23]. In brief, a quantitative β-galactosidase activity using ONPG was quantified with the formula 1000 × OD_420_/(OD_600_ × assay time in min × assay volume in mL). β-Galactosidase filter assay was conducted using 5-bromo-4-chloro-3-indolyl-β-d-galactopyranoside as a substrate for 6 h. Yeast transformants grown on SD media lacking Trp and Ura were incubated for 3–5 days at 30 °C for the growth assay.

### 4.7. Dual-luciferase Assay

Firefly luciferase and Nano luciferase activities were quantified using the GloMax®-Multi+ Detection System (Promega Corp., Madison, WI, USA) with Instinct^TM^ Software and Nano-Glo® Dual-Luciferase® Reporter Assay System (Promega Corp., Madison, WI, USA).

### 4.8. RNA Isolation and RT-PCR

Total RNA was isolated using the RNAqueous RNA Isolation Kit (Invitrogen, Carlsbad, CA, USA) and Plant RNA Isolation Aid (Invitrogen, Carlsbad, CA, USA), in accordance with the manufacturer’s protocol. Total RNA (2 μg) was used for reverse transcription using Moloney murine leukemia virus reverse transcriptase (Promega Corp., Madison, WI, USA) as previously described [23].

RT-qPCR was conducted using a QuantStudio^TM^ 3 real-time PCR system (Applied Biosystems, Foster, CA, USA) and Power SYBR^TM^ Green PCR Master Mix (Applied Biosystems, Foster, CA, USA) in accordance with manufacturer’s manual. Real-time DNA amplification was analyzed using QuantStudio^TM^ Design and Analysis software (version 1.4.3) (Applied Biosystems, Foster, CA, USA). Three independent reactions were conducted for each technical replicate. Two technical replicates were conducted for each biological replicate.

Semi-quantitative RT-PCR was conducted in accordance with previous study [23]. PCR reactions were repeated 30–31 cycles for *AtC3H12* and 23–24 cycles for *GAPc*.

Primers for RT-PCR are shown in Table S2.

### 4.9. GUS Assay

Histochemical GUS assay was performed in accordance with the method described previously [23].

### 4.10. Phenotype Analysis

To measure the germination percentage, FW, and primary root length, 30 seeds of each plant were sown on the same MS agar plate and grown under SD conditions. Germination was determined by radicle protrusion. Primary root length was measured using ImageJ [41]. At least three biological replicates were performed.

### 4.11. Statistical Analysis

Statistical analysis was performed by IBM SPSS Statistics software version 23 (IBM Corp., Armonk, NY, USA) with one-way ANOVA using Tukey’s multiple comparison test.

## Data Availability

The data presented in this study are available in the Supplementary Materials provided here.

## Supplementary Materials

The following supporting information can be downloaded at: https://www.mdpi.com/article/10.3390/ijms23031572/s1.
