# Supplementary material for: Non-TZF Transcriptional Activator AtC3H12 Negatively Affects Seed Germination and Seedling Development in Arabidopsis
Source: Int J Mol Sci. 2022 Jan 29;23(3):1572. doi: 10.3390/ijms23031572 (PMC8835867; doi:10.3390/ijms23031572)
Supplement: Supplementary file 1 [file ijms-23-01572-s001.zip › ijms-1537960-supplementary.pdf]

Supplementary data

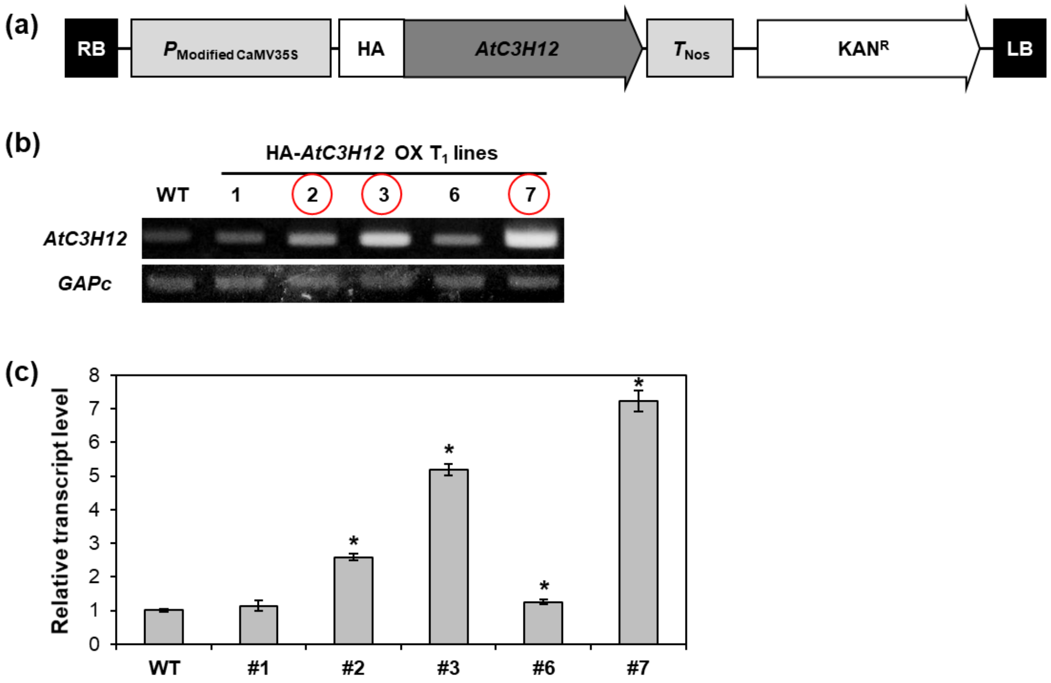

Figure S1. Selection of *AtC3H12* OXs. (**a**) Schematic map of the vector for overexpression of *AtC3H12*. (**b**) Transcript levels of *AtC3H12* in WT and *AtC3H12* OX T_1 lines determined by semi-RT-qPCR. Circled lines were selected for further analysis. The representative results came from at least three reactions. (**c**) Transcript levels of *AtC3H12* in WT and *AtC3H12* OX T_1 lines were determined by RT-qPCR. Transcript level in WT was set as 1. At least two biological replicates showed similar results. Error bars display standard deviation ($n = 3$) and * display t-test $P < 0.05$. In (**b**) and (**c**), *GAPc* was used for an endogenous control gene.

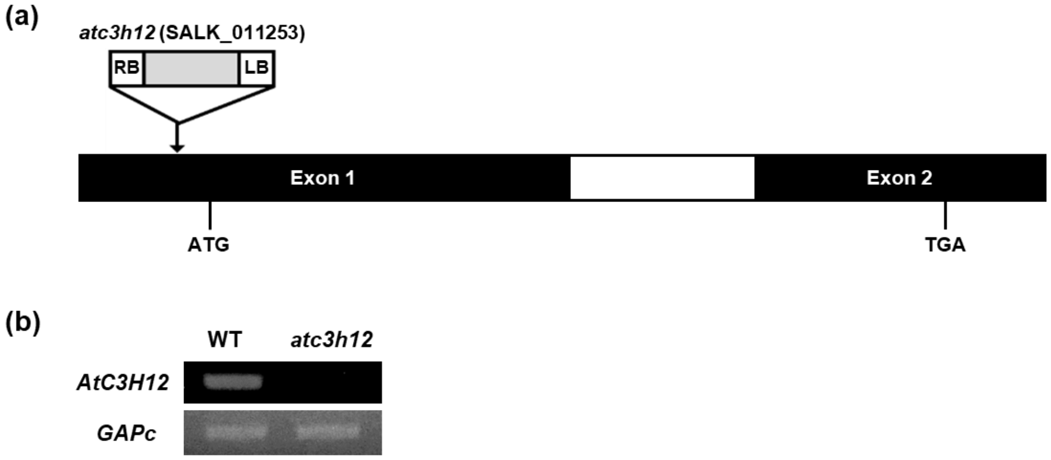

Figure S2. Confirmation of *atc3h12* mutant. (**a**) Genomic structure of the *AtC3H12* locus is shown. The black and white boxes represent the exon and intron, respectively. T-DNA insertion site of *atc3h12* mutant (SALK_011253) was drawn with an arrow. (**b**) Semi-RT-qPCR analysis of *AtC3H12* in WT and *atc3h12* seedlings at 14 DAG. *GAPc* was used for an endogenous control gene.

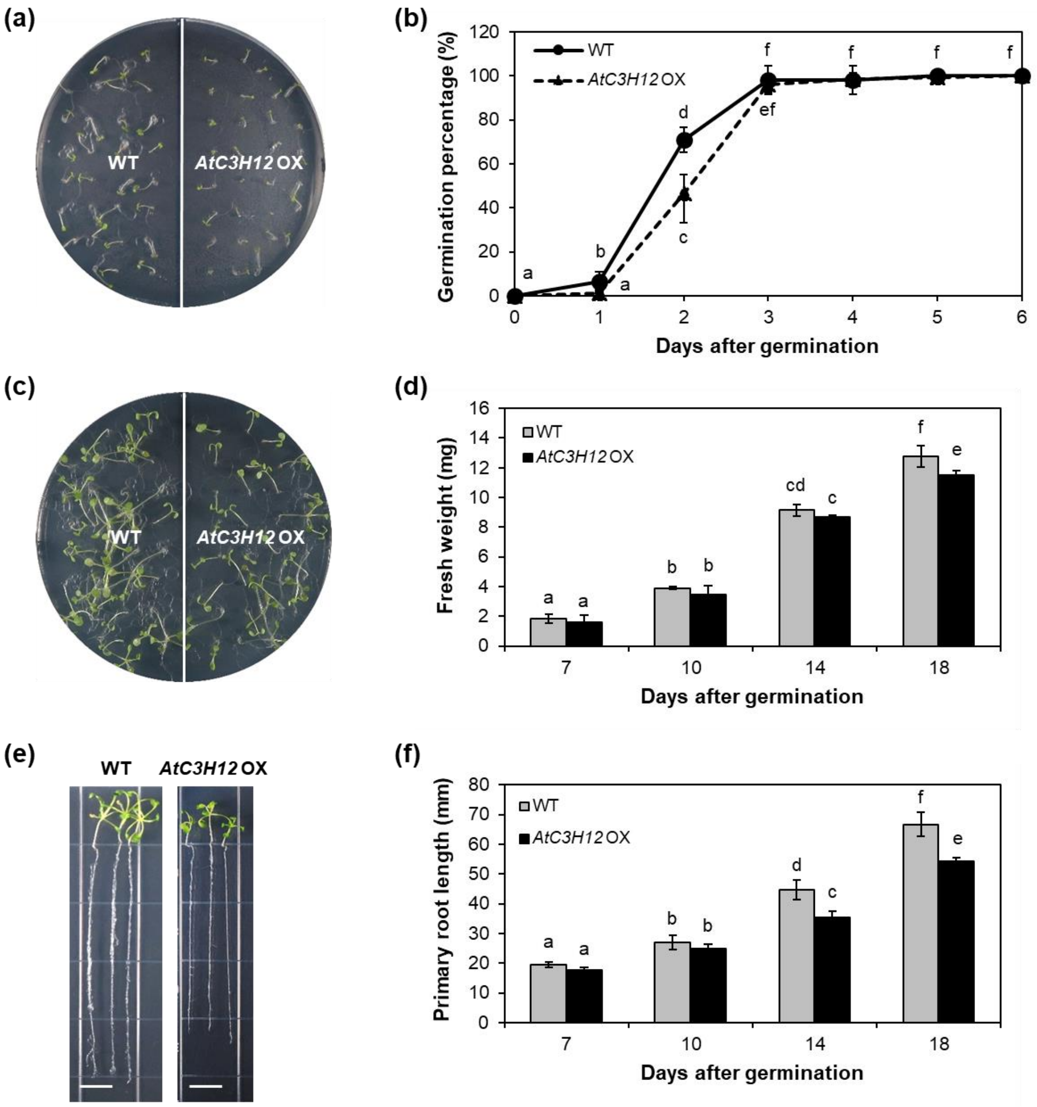

Figure S3. Seed germination and seedling development of *AtC3H12* OXs. (**a**) Four-day-old WT and *AtC3H12* OX-3 grown on MS agar plates under SD conditions. (**b**) Germination percentage of WT and *AtC3H12* OX-3 measured at specified times after sowing on MS agar plates. Germination was verified by radicle protrusion. Error bars display standard deviation (n = 20). (**c**) Ten-day-old WT and *AtC3H12* OX-3 seedlings grown on MS agar plates under SD conditions. (**d**) FW of shoots of WT and *AtC3H12* OX-3 seedlings grown on MS agar media at 7, 10, 14, and 18 DAG. Error bars display standard deviation (n = 5). (**e**) Elongation of primary roots of WT and *AtC3H12* OX-3

seedlings at 18 DAG. (**f**) Primary root lengths of WT and *AtC3H12* OX-3 seedlings grown on MS agar plates under SD conditions were measured at 7, 10, 14, and 18 DAG. Error bars display standard deviation (n = 10). In (**b**), (**d**) and (**f**), different letters display significant differences ($p < 0.05$). In (**e**), the white line indicates scale bar = 1 cm. *AtC3H12* OX-2 showed similar results.

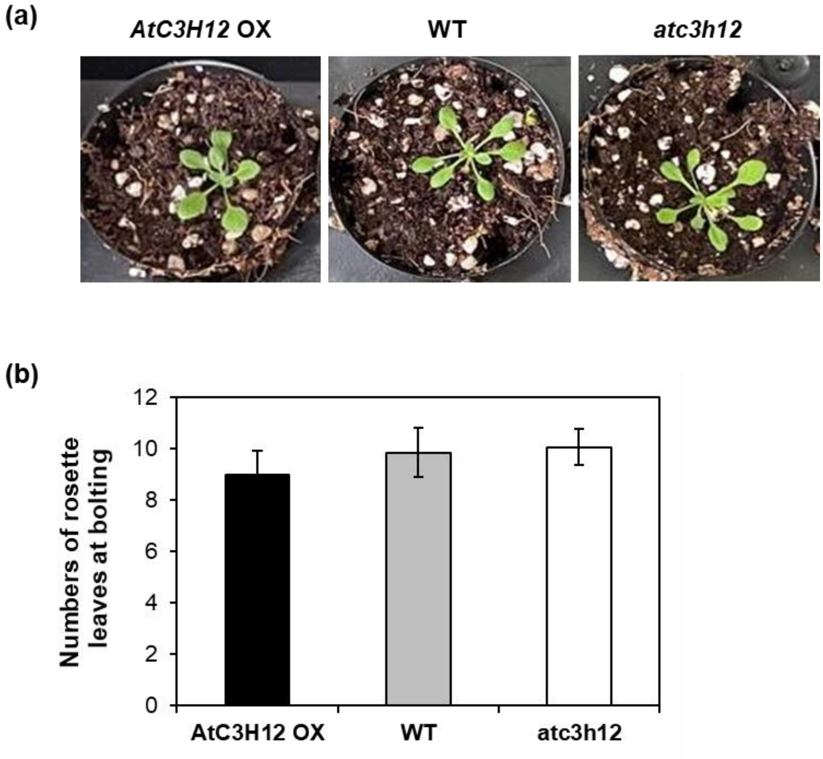

Figure S4. Flowering time of *AtC3H12* OXs and *atc3h12* mutants. (**a**) Sixteen-day-old WT, *AtC3H12* OX, and *atc3h12* mutants grown on soil under LD conditions. WT, *AtC3H12* OX, and *atc3h12* plants were simultaneously sown and grown. (**b**) The number of rosette leaves of WT, *AtC3H12* OX, and *atc3h12* plants at bolting. Error bars display standard deviation (n = 15).

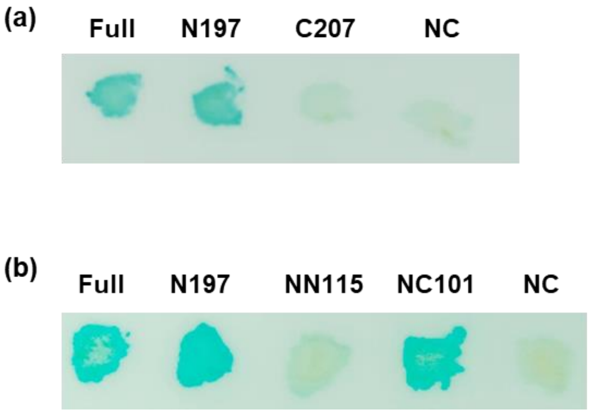

Figure S5. Transactivation activity assay of AtC3H12 in yeast. (**a**) β-Galactosidase filter assay of full-length ORF, N197, and C207 of AtC3H12. (**b**) β-Galactosidase filter assay of full-length ORF, N197, NN115, and NC101 of AtC3H12. In (**a**) and (**b**), yeast transformants were incubated with X-gal solutions at 30℃ for 6 h. The empty pBD-GAL4 vector was used for a negative control. NC, negative control.

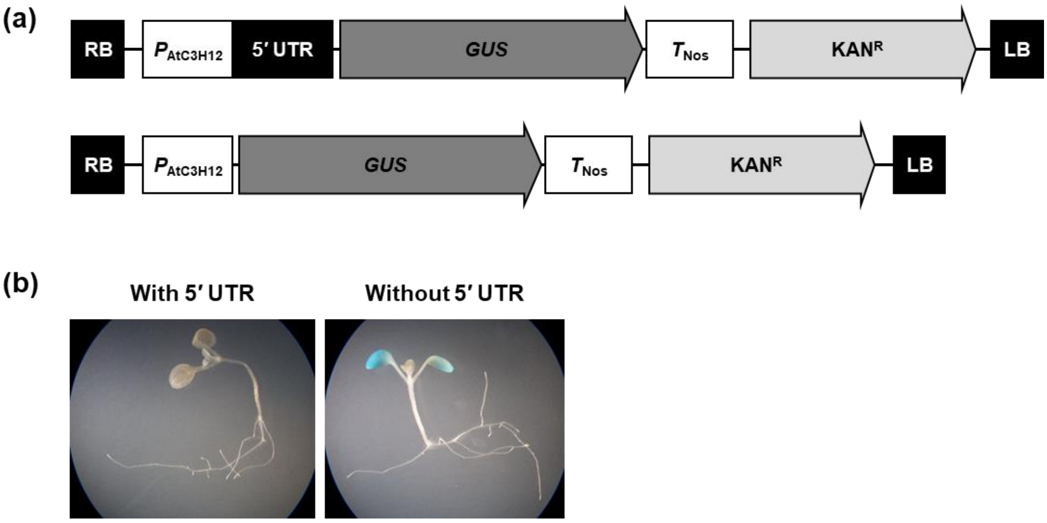

Figure S6. Comparison of the promoter activity of *AtC3H12*. (**a**) Schematic maps of P_{AtC3H12}::*GUS* constructs with and without 5′ UTR. (**b**) Histochemical assay of *GUS* expression levels in transgenic plants carrying P_{AtC3H12}::*GUS* with and without 5′ UTR at 11 DAG. Representative GUS staining results are presented here.

Table S1. List of primers for cloning

Construct	Forward	Reverse
AtC3H12 OX	5′-AATGTCGACATGAGTCATCACCGGCGAGA-3′	5′-GCGGGATCCTCATTCAATATCGTCGATCC-3′
sGFP-AtC3H12	5′-AATGTCGACATGAGTCATCACCGGCGAGA-3′	5′-GCGGGATCCTCATTCAATATCGTCGATCC-3′
AtC3H12-sGFP	5′-AATGTCGACATGAGTCATCACCGGCGAGA-3′	5′-GCACCCGGGTTCAATATCGTCGATCCAAT-3′
$P_{AtC3H12}$::*GUS* (without 5′ UTR)	5′-CGCCCCGGGTTTGAGTTTTTTTTAATAATAC-3′	5′-CGCGTCGACTACAGTGTATTAAATGATCA-3′
$P_{AtC3H12}$::*GUS* (with 5′ UTR)	5′-CGCCCCGGGTTTGAGTTTTTTTTAATAATAC-3′	5′-CGCGTCGACCGTGTGAAAAATCGAAGCTT-3′
For transactivation analysis in yeast		
AtC3H12 full-length ORF	5′-ATCGAATTCATGAGTCATCACCGGCGAGA-3′	5′- CGCGTCGACTCATTCAATATCGTCGATCC-3′
AtC3H12 N197	5′-ATCGAATTCATGAGTCATCACCGGCGAGA-3′	5′-CGCGTCGACTCAATGCAGAAACGTACAACTTT-3′
AtC3H12 C207	5′-CGCGAATTCTGTAAGAAGTTTTATACTGA-3′	5′- CGCGTCGACTCATTCAATATCGTCGATCC-3′
AtC3H12 NN115	5′-ATCGAATTCATGAGTCATCACCGGCGAGA-3′	5′-CGCGTCGACTCAATGAGCAAAGTTACAATTCG-3′
AtC3H12 NC101	5′-ATAGAATTCTGTTGCAAGTTTCGTGCTGG-3′	5′-CGCGTCGACTCAATGCAGAAACGTACAACTTT-3′
For transactivation assay in Arabidopsis protoplasts		
AtC3H12 full-length ORF	5′-AGTTCTAGAATGAGTCATCACCGGCGAGA-3′	5′-AGTCCCGGGTCATTCAATATCGTCGATCC-3′
AtC3H12 N197	5′-AGTTCTAGAATGAGTCATCACCGGCGAGA-3′	5′-CGCCCCGGGTCAATGCAGAAACGTACAACTTT-3′
AtC3H12 NC101	5′-CACTCTAGATGTTGCAAGTTTCGTGCTGG-3′	5′-CGCCCCGGGTCAATGCAGAAACGTACAACTTT-3′

Table S2. List of primers for RT-qPCR

Gene	Forward	Reverse	Purpose
GAPc	5'-GTGTCCCAACCGTTGATGTC-3'	5'-TCCCTTGAGTTTGCCTTCGG-3'	Quantitative RT-PCR
AtC3H12	5'-TCATACGGTGGAGGAGCTTC-3'	5'-ACTGTAGGAGTTCCCATACC-3'	
GAPc	5'-CACTTGAAGGGTGGTGCCAAG-3'	5'-CCTGTTGTCGCCAACGAAGTC-3'	Semi-quantitative RT-PCR
AtC3H12	5′-TGTTTGGGCGACGGAAGATG-3′	5′-ACTGTAGGAGTTCCCATACC-3′	
